# Prognostic models for COVID-19 needed updating to warrant transportability over time and space

**DOI:** 10.1186/s12916-022-02651-3

**Published:** 2022-11-23

**Authors:** David van Klaveren, Theodoros P. Zanos, Jason Nelson, Todd J. Levy, Jinny G. Park, Isabel R. A. Retel Helmrich, Judith A. C. Rietjens, Melissa J. Basile, Negin Hajizadeh, Hester F. Lingsma, David M. Kent

**Affiliations:** 1 Department of Public Health, Erasmus MC University Medical Center Rotterdam, Dr. Molewaterplein 50, 3015 GE Rotterdam, The Netherlands; 2 Predictive Analytics and Comparative Effectiveness Center, Institute for Clinical Research and Health Policy Studies, Tufts Medical Center, Boston, USA; 3 Institute of Bioelectronic Medicine, Feinstein Institutes for Medical Research, Northwell Health, Manhasset, NY USA; 4 Division of Pulmonary Critical Care and Sleep Medicine, Department of Medicine, Donald and Barbara Zucker School of Medicine at Hofstra/Northwell Health, Hempstead, NY USA

**Keywords:** COVID-19, Emergency department, Clinical prediction models, Generalizability, Transportability

## Abstract

**Background:**

Supporting decisions for patients who present to the emergency department (ED) with COVID-19 requires accurate prognostication. We aimed to evaluate prognostic models for predicting outcomes in hospitalized patients with COVID-19, in different locations and across time.

**Methods:**

We included patients who presented to the ED with suspected COVID-19 and were admitted to 12 hospitals in the New York City (NYC) area and 4 large Dutch hospitals. We used second-wave patients who presented between September and December 2020 (2137 and 3252 in NYC and the Netherlands, respectively) to evaluate models that were developed on first-wave patients who presented between March and August 2020 (12,163 and 5831). We evaluated two prognostic models for in-hospital death: The Northwell COVID-19 Survival (NOCOS) model was developed on NYC data and the COVID Outcome Prediction in the Emergency Department (COPE) model was developed on Dutch data. These models were validated on subsequent second-wave data at the same site (temporal validation) and at the other site (geographic validation). We assessed model performance by the Area Under the receiver operating characteristic Curve (AUC), by the E-statistic, and by net benefit.

**Results:**

Twenty-eight-day mortality was considerably higher in the NYC first-wave data (21.0%), compared to the second-wave (10.1%) and the Dutch data (first wave 10.8%; second wave 10.0%). COPE discriminated well at temporal validation (AUC 0.82), with excellent calibration (E-statistic 0.8%). At geographic validation, discrimination was satisfactory (AUC 0.78), but with moderate over-prediction of mortality risk, particularly in higher-risk patients (E-statistic 2.9%). While discrimination was adequate when NOCOS was tested on second-wave NYC data (AUC 0.77), NOCOS systematically overestimated the mortality risk (E-statistic 5.1%). Discrimination in the Dutch data was good (AUC 0.81), but with over-prediction of risk, particularly in lower-risk patients (E-statistic 4.0%). Recalibration of COPE and NOCOS led to limited net benefit improvement in Dutch data, but to substantial net benefit improvement in NYC data.

**Conclusions:**

NOCOS performed moderately worse than COPE, probably reflecting unique aspects of the early pandemic in NYC. Frequent updating of prognostic models is likely to be required for transportability over time and space during a dynamic pandemic.

**Supplementary Information:**

The online version contains supplementary material available at 10.1186/s12916-022-02651-3.

## Background

The coronavirus disease (COVID-19) pandemic has been characterized by a high uncertainty in outcomes for those contracting the virus, particularly regarding the severity of symptoms, disease trajectories, and mortality. Additionally, there are differences in governmental public health responses between countries and between surges in COVID-19 cases (“waves”) [1]. As such, outcomes have varied by geographic region and temporally by “wave” [2]. This has further exacerbated uncertainty, making it difficult to predict outcomes among people with COVID-19 who are admitted to the hospital.

Approximately 20% of patients hospitalized with COVID-19 require intensive care and possibly invasive mechanical ventilation [3, 4]. Patient preferences with COVID-19 for mechanical ventilation may be different than for other types of pneumonia, because intubation for these patients is often prolonged, may be administered in settings characterized by severe social isolation and is associated with very high average mortality rates [3, 5]. Supporting patients and surrogate decision-makers in conversations facing decisions regarding admission to the intensive care unit (ICU) and mechanical ventilation requires providing an accurate forecast of their likely outcomes based on their individual characteristics [3, 6]. Further, given the continuous pressure on health care systems, there is a need to support decision-making in triaging people with COVID-19 in the Emergency Department (ED) for hospital or ICU admission. Clinical prediction models have the potential to support health care providers and people with COVID-19 and their families in decision-making by providing accurate prognoses.

Since the start of the pandemic, over 200 prediction models for the diagnosis and prognosis of COVID-19 have appeared in the literature, but few were developed with high methodological rigor [7]. Almost all published models were identified as having a high risk of bias, indicating that their reported performance is most likely overly optimistic [7]. Although some of these models were externally validated — showing highly variable model performance — the validity and generalizability in settings beyond those in which the model was developed remains largely unknown [8]. Poorly calibrated prognostic models may lead to harm, since they yield misinformation that can lead to clinical decision-making that is worse than using best “on average” information [9–11].

In addition to examining geographic transportability, since new SARS-CoV-2 variants with different COVID-19 severity are emerging (such as Omicron), natural and vaccine immunity are developing and treatment best practices are rapidly evolving over time (e.g., proning, minimizing paralytics, lung-protective volumes, remdesivir, dexamethasone), validating and updating these prognostic models may be crucial, even within the same geographic setting [12, 13]. Changes over time in the selection of patients who are admitted to the hospital can also have important effects on outcomes and on the consistency of predictor effects. Models developed on abundant first-wave data may have little generalizability to later waves.

Both in the New York City (NYC) area and the Netherlands (NL), prognostic models were developed for predicting outcomes in patients hospitalized with COVID-19: The Northwell COVID-19 Survival (NOCOS) model was developed on a large set of NYC data and the COVID Outcome Prediction in the Emergency Department (COPE) model was developed on a large set of NL data [14, 15]. We aimed to evaluate the geographic and temporal transportability of these two models and to examine updating approaches. Thus, we sought to gain further insight on model transportability to different settings and to different time windows, particularly in a dynamic pandemic.

## Methods

### Population

The database included anonymized data of COVID-19 patients who were admitted to 12 Northwell Clinics in the NYC area and to 4 Dutch hospitals. NOCOS and COPE were developed on data of patients who presented at the ED and were admitted to the hospital with suspected COVID-19 in the first wave of the pandemic between March and August 2020, in NYC and NL respectively. To evaluate the temporal and geographic transportability of NOCOS and COPE, we used data of patients who presented at the ED and were admitted to the hospital with suspected COVID-19 in the second wave of the pandemic, between September and December 2020. Patients being transferred to other hospitals were excluded since information on outcomes was missing.

### Outcomes

The outcomes of interest were (a) death or transfer to a hospice within 28 days after hospital admission and (b) requiring mechanical ventilation (NYC) or ICU admission (NL) within 28 days after hospital admission.

### Predictors

Based on prior literature both NOCOS and COPE included patient characteristics (sex, age, BMI), vital parameters (oxygen saturation, systolic blood pressure, heart rate, respiratory rate [RR], body temperature), and blood test values (C-reactive protein [CRP], lactic dehydrogenase [LDH], D-Dimer, leucocytes, lymphocytes, monocytes, neutrophils, eosinophils, Mean Corpuscular Volume [MCV], albumin, bicarbonate, sodium, creatinine, urea), all measured at ED admission [7, 14, 15]. Logarithmic transformations of predictor values were included to capture non-linear associations with the outcomes. The date of admission was included to capture potential secular changes in outcomes over time; these variables were fixed to calibrate risk predictions to outcome rates at the end of the first wave. In the case of multiple measurements for the same patient, we used the first measurement after presentation at the ED. We used Multivariate Imputation by Chained Equations (R-packages mice) for multiple imputation of missing predictor values [16, 17]. Multiple imputation in the validation data was undertaken separately from multiple imputation in the development data to ensure fully independent model validation.

### Model development

Details on the development of COPE and NOCOS are described in other publications [14, 15]. A summary of important details is provided in the supplement (Additional file 1: Box S1), together with the model formulas (Additional file 1: Table S1).

### Model validation

Model performance was assessed temporally on subsequent second-wave data at the same site and also geographically, i.e., COPE was evaluated on second-wave NYC data and NOCOS on second-wave NL data. We assessed discriminative ability with the area under the operator receiver characteristic curve (AUC). The model-based concordance (mb.c), which provides the expected AUC in a validation dataset based on the distribution of the predicted probabilities (i.e., assuming no model invalidity), was used to understand the impact on the discriminative ability of potential differences in case-mix heterogeneity between the development and validation data [18]. We assessed calibration with calibration plots of ten equally sized groups of predicted risk, with the E-statistic — the average absolute difference between predicted probabilities and observed frequencies according to a smooth calibration curve – and with calibration intercepts and calibration slopes [19]. We used decision curves to assess the net benefit of using the models at a range of decision thresholds [20]. We also evaluated the net benefit after updating the intercept and the slope in the validation data. All analyses were performed in MATLAB 2019b and in R software, at the NYC and Dutch site, respectively [16].

## Results

### Patient characteristics and outcomes

#### Mortality

Twenty-eight-day mortality was considerably higher in the NYC first-wave data (2551/12,163 = 21.0%), compared to the second-wave (216/2137 = 10.1%) and the NL data (first wave 629/5831 = 10.8%; second wave 326/3252 = 10.0%). Many predictors were similarly distributed in the NL and the NYC area, with the exception of CRP and LDH, which were higher, and D-Dimer, which was lower in the NYC area (Table 1). These biomarkers may have been measured in sicker patients, reflected in higher biomarker levels when larger proportions were missing.

**Table 1:** Baseline characteristics of 1st wave and 2nd wave patient cohorts in the Netherlands and NYC. Median, quartile range (“Q1” = first quartile; “Q3” = third quartile) and percentage missing (“% NA”) are presented for all continuous variables. The percentage of patients with male sex is reported in the last row

	NL 1st wave ***n*** = 5831				NL 2nd wave ***n*** = 3252				NYC 1st wave ***n*** = 12,163				NYC 2nd wave ***n*** = 2137			
	***% NA***	***Median***	***Q1***	***Q3***	***% NA***	***Median***	***Q1***	***Q3***	***% NA***	***Median***	***Q1***	***Q3***	***% NA***	***Median***	***Q1***	***Q3***
Age (years)	0	70	58	80	0	71	58	80	0	65	54	77	0	66	54	77
BMI (kg/m^2^)	58	26	23	30	59	26	23	30	17	28	24.6	32.3	2	28.1	24.9	32.4
HR (bpm)	39	90	78	103	40	90	78	105	3	90	79	102	0	87	76	98
SBP (mmHg)	42	133	118	150	43	134	119	151	3	127	114	143	0	129	115	145
RR (/min)	42	19	16	23	43	20	16	24	3	20	18	23	0	20	18	22
Saturation (%)	41	95.8	94.0	97.5	40	95.7	94.0	97.5	3	97	95	99	0	97	95	99
Temperature (°C)	40	37.3	36.7	38.1	42	37.3	36.7	38.1	4	37.2	36.8	37.9	1	37.1	36.7	37.7
CRP (mg/L)	7	48	10	118	9	57	16	124	41	109	56	186	6	74	34	131
D-Dimer (μg/L)	64	1100	527	2545	76	1060	531	2170	36	477	282	1033	5	307	200	509
LDH (U/L)	18	244	200	322	22	247	203	334	41	431	329	568	9	354	277	461
Leucocytes (×10^9/L)	7	9.1	6.7	12.7	10	9.4	6.6	12.9	3	7.6	5.6	10.4	1	6.9	5.2	9.4
Lymphocytes (x10^9/L)	16	1.04	0.66	1.6	20	0.98	0.62	1.50	4	0.92	0.64	1.33	1	0.91	0.63	1.35
Albumin (g/L)	15	39	35.5	42	20	39	35	42	3	34	30	38	1	36	32	40
Bicarbonate (mmol/L)	45	23.6	21	26	50	23.5	21	26	3	24	21	26	1	24	22	26
Creatinine (μmol/L)	8	84	66	111	10	84	66	116	3	93	72	133	1	88	71	121
Eosinophils (×10^9/L)	26	0.03	0.00	0.10	27	0.03	0.01	0.10	5	0	0	0.04	1	0.01	0	0.05
MCV (fL)	7	90	87	94	10	90	87	94	5	88	84	92	1	88	85	92
Monocytes (×10^9/L)	30	0.67	0.44	0.95	30	0.67	0.43	0.98	4	0.49	0.33	0.71	1	0.48	0.33	0.69
Neutrophils (×10^9/L)	16	5.6	2.2	9.0	21	5.8	2.4	9.4	4	5.9	4.1	8.5	1	5.1	3.6	7.4
Sodium (mmol/L)	9	138	135	140	11	137	134	139	3	136	133	139	1	136	134	139
Urea (mmol/L)	9	6.5	4.6	9.7	11	6.9	4.9	10.4	3	6.4	4.3	11.1	1	6.1	4.3	9.3
Male sex	0	57%			0	56%			0	57%			0	57%		

#### Need for mechanical ventilation or ICU admission

In the NYC area, the proportion of patients receiving mechanical ventilation decreased from 16.9% (2056/12,163) in the first wave to 10.4% (223/2135) in the second wave of the pandemic. The rate of ICU admission in NL (fully recorded for two out of four hospitals) decreased from 8.1% (214/2633) in the first wave to 5.9% (86/1466) in the second wave of the pandemic. However, for validation of the models predicting the need of mechanical ventilation or ICU admission in the NL data, we only used patients below the age of 70, as the probability of being admitted to the ICU paradoxically decreased with age after the age of 70, reflecting a triage policy not to admit older patients to the ICU rather than using a triage policy based on disease severity [15]. The rate of ICU admission in patients below the age of 70 in NL decreased from 9.9% (128/1296) in the first wave to 6.4% (45/706) in the second wave of the pandemic.

### Validation of prognostic models

#### Mortality

**Fig. 1:**
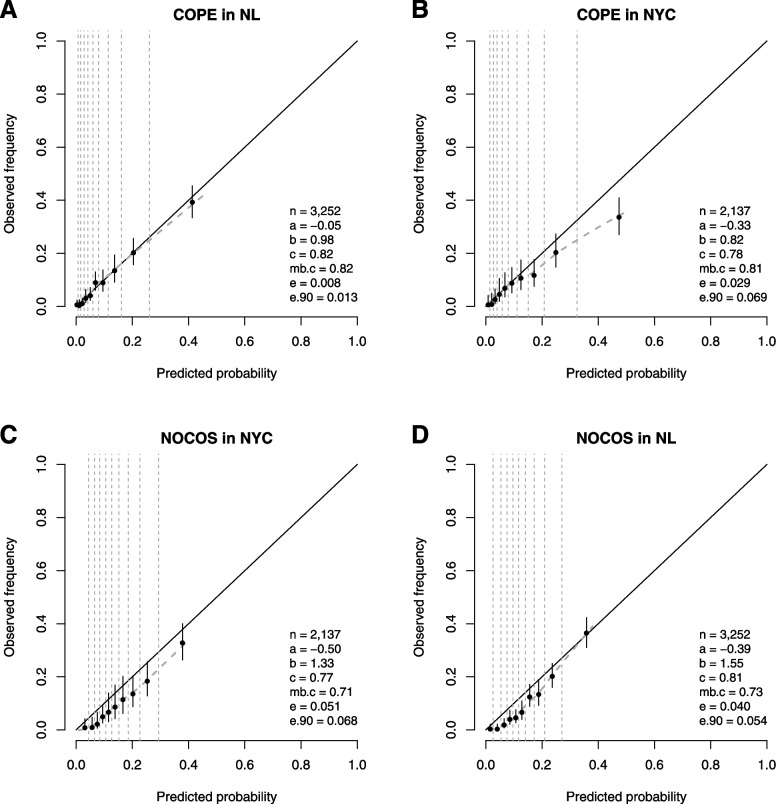
Temporal and geographic validation: performance of COPE and NOCOS for predicting death in second-wave patients. Calibration plots of patients who were admitted since September 2020 in 4 NL hospitals and 12 NYC hospitals. Temporal validations of COPE and NOCOS are in panels **A** and **C**, respectively. Geographic validations of COPE and NOCOS are in panels **B** and **D**, respectively. n is number of patients; a = calibration intercept (0 is perfect); b = calibration slope (1 is perfect); c = AUC (0.5 is useless; 1 is perfect); mb.c = model-based AUC

COPE discriminated well at temporal validation (AUC 0.82 [0.80; 0.84]; Fig. 1A), with excellent calibration (E-statistic 0.8%; calibration intercept −0.05 [−0.17; 0.08]; calibration slope 0.98 [0.86; 1.10]). At geographic validation in second-wave NYC data, discrimination was satisfactory (AUC 0.78 [0.75; 0.81]; Fig. 1B), but with moderate over-prediction of mortality risk, particularly in higher risk patients (E-statistic 2.9%; calibration intercept −0.33 [−0.50; −0.17]; calibration slope 0.82 [0.66; 0.97]).

In contrast, when NOCOS was evaluated in NYC area data from the second wave, while discrimination was adequate (AUC 0.77 [0.74; 0.81]; Fig. 1C), NOCOS systematically overestimated the mortality risk (E-statistic 5.1%; calibration intercept −0.50 [−0.65; −0.34]). Similarly, when tested in NL data, discrimination remained adequate (AUC 0.81 [0.79; 0.83]; Fig. 1D), but again NOCOS over-predicted mortality risk, particularly in lower risk patients (E-statistic 4.0%; calibration intercept −0.39 [−0.51; −0.28]). Surprisingly, NOCOS was “underfitted” (calibration slope>1), both at temporal validation (calibration slope 1.33 [1.10; 1.57]) and geographic validation (calibration slope 1.55 [1.36; 1.74]), probably due to overly aggressive shrinkage of the predictor effects of NOCOS.

**Fig. 2:**
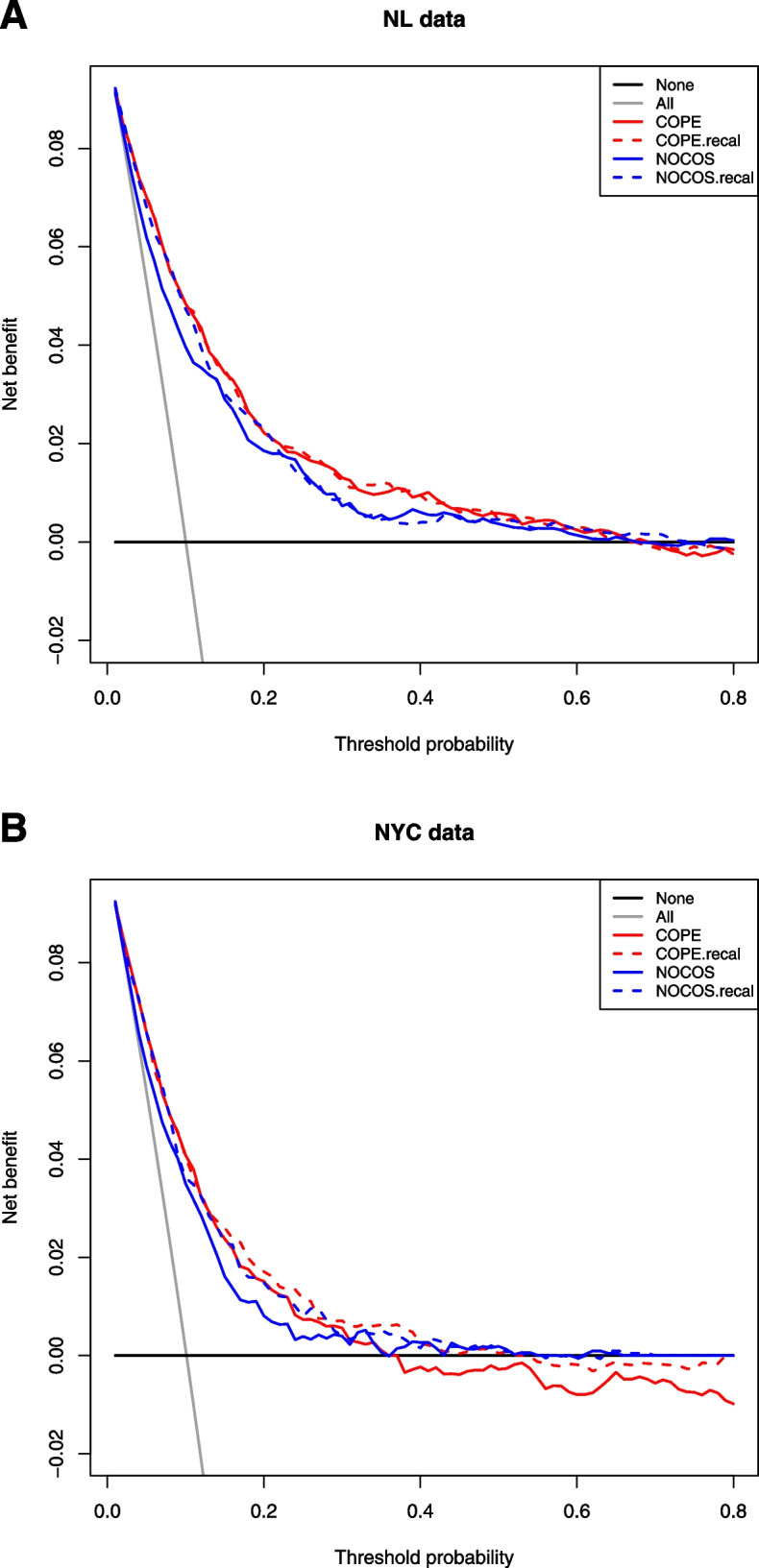
Temporal and geographic validation: net benefit of COPE and NOCOS for predicting death in second-wave patients. Decision curves of patients who were admitted since September 2020 in 4 NL hospitals (panel **A**) and 12 NYC hospitals (panel **B**). Net benefit is plotted against the full range of possible decision threshold probabilities for the original prognostic models (“COPE” in red and “NOCOS” in blue) and for these models with a calibrated intercept and slope (“COPE.recal” in dashed red and “NOCOS.recal” in dashed blue)

In NL data, both COPE and NOCOS had a positive net benefit for decision thresholds up to 70%, but the net benefit of COPE was considerably higher for decision thresholds up to 50% (Fig. 2A). Recalibration of both models to the second-wave NL data led to limited improvements in net benefit. In the NYC data, the net benefit of COPE was positive and more favorable than the net benefit of NOCOS for decision thresholds up to 30%, but was negative for decision thresholds over 35%, while NOCOS was not negative for the full range of decision thresholds. After recalibration of the intercept and the slope to the second-wave NYC data (Fig. 2B), the net benefit of COPE and NOCOS was more similar.

#### Exploring the influence of changes in outcome rates over time in the first wave

To explore variations in outcome rates over time, we examined COPE and NOCOS predictions when the variable for calendar time was excluded from the model and compared that to performance of the full model. When NL data from March was used to predict outcome rates in the second-wave NL data, the average predicted mortality was 17.1%, an over-prediction of 7.1%. When data from the full first wave was used, the average predicted mortality in the second wave decreased to 12.3%, an over-prediction of 2.3%. Correcting for the “March effect” led to the excellent calibration, with an average over-prediction of only 0.5%. The NOCOS model developed on first-wave data from March also over-predicted mortality in the second-wave NYC data; the average predicted mortality was 17.3%, an over-prediction of 7.1%. However, using NYC data from the full first wave or including a time effect did not correct this over-prediction; these models yielded over-predictions of 7.2% and 5.2%, respectively.

#### Need for mechanical ventilation or ICU admission

**Fig. 3:**
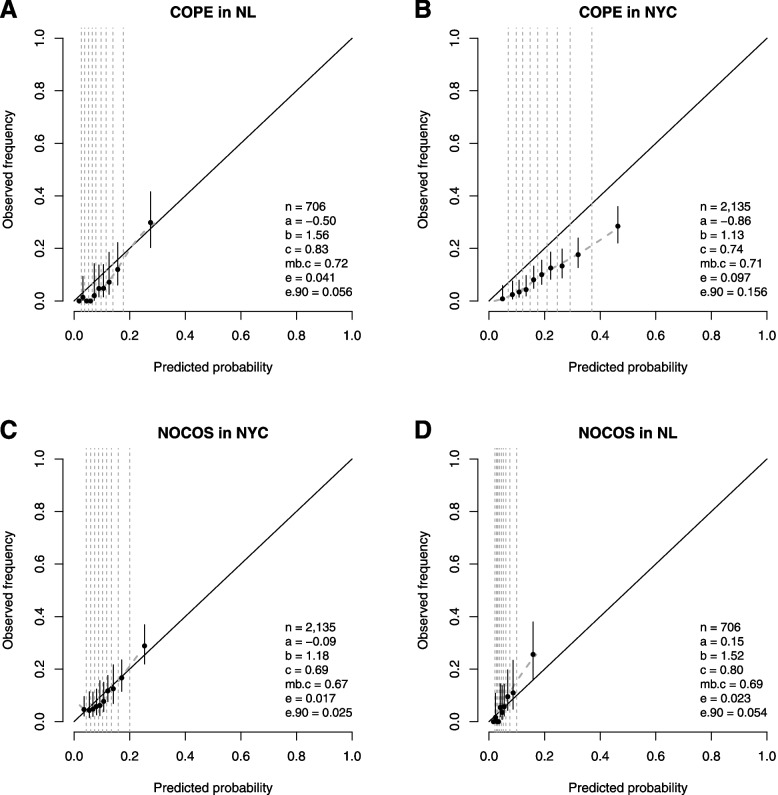
Temporal and geographic validation: Performance of COPE and NOCOS for predicting the need for ICU admission (NL) or mechanical ventilation (NYC) in second-wave patients. Calibration plots of patients who were admitted since September 2020 in 4 NL hospitals and 12 NYC hospitals. Temporal validations of COPE and NOCOS are in panels A and C, respectively. Geographic validations of COPE and NOCOS are in panels B and D, respectively. n is number of patients; a = calibration intercept (0 is perfect); b = calibration slope (1 is perfect); c = AUC (0.5 is useless; 1 is perfect); mb.c = model-based AUC

Although COPE significantly over-predicted ICU admission in second-wave patients in NL (Fig. 3A; calibration intercept −0.50 [−0.81; −0.19]; E-statistic 4.1%), it was well able to identify the patients at high risk of needing ICU admission, as expressed by good discriminative ability (AUC 0.83 [0.78; 0.89]) and substantially stronger predictor effects than in the development data (calibration slope 1.56 [1.11; 2.01]). COPE also substantially over-predicted the need for mechanical ventilation in NYC, possibly because it was designed to predict the need for ICU admission rather than the need for mechanical ventilation (Fig. 3B; calibration intercept −0.86 [−1.00; −0.71]; E-statistic 9.7%), but the discriminative ability (AUC 0.74 [0.71; 0.77]) was more in line with expectations (mb.c 0.71) and the calibration slope (1.13 [0.90; 1.37]) was much closer to ideal (slope 1).

NOCOS was well calibrated (Fig. 3C; calibration intercept −0.09 [−0.24; 0.07]; calibration slope 1.18 [0.85; 1.50]; E-statistic 1.7%) with a discriminative performance similar to expectation in second-wave NYC patients (AUC 0.69 [0.65; 0.74] versus mb.c 0.67). NOCOS predicted the need for ICU admission in NL very well on average (Fig. 3D; calibration intercept 0.15 [−0.16; 0.45]; E-statistic 2.3%), but the predictor effects were significantly stronger in NL (calibration slope 1.52 [1.05; 2.00]), also reflected by the much better discriminative ability than expected (AUC 0.80 [0.74; 0.87] versus mb.c 0.69).

**Fig. 4:**
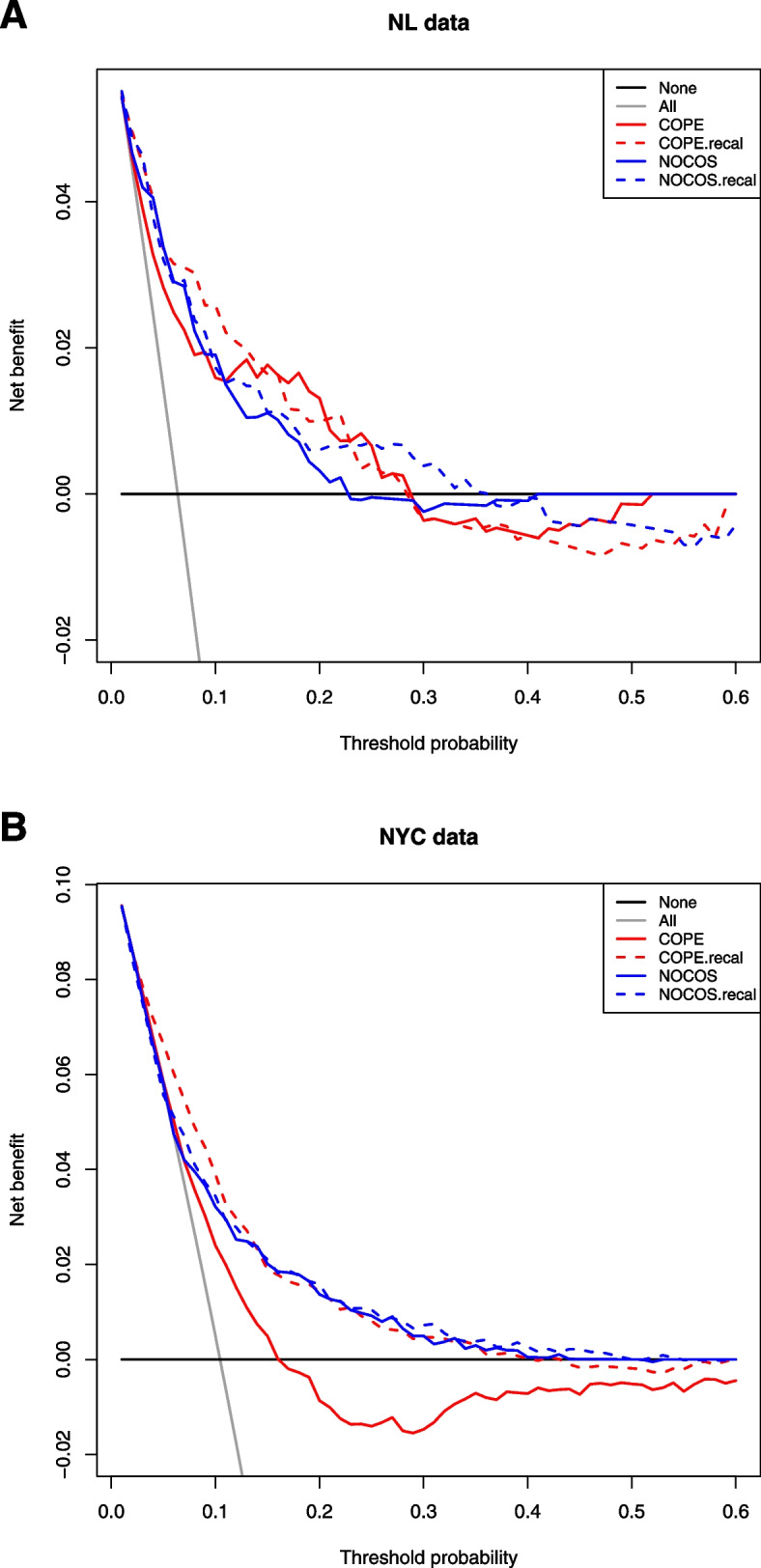
Temporal and geographic validation: Net benefit of COPE and NOCOS for predicting need for ICU admission (NL) or mechanical ventilation (NYC) in second-wave patients. Decision curves of patients who were admitted since September 2020 in 4 NL hospitals (panel **A**) and 12 NYC hospitals (panel **B**). Net benefit is plotted against the full range of possible decision threshold probabilities for the original prognostic models (“COPE” in red and “NOCOS” in blue) and for these models with a calibrated intercept and slope (“COPE.recal” in dashed red and “NOCOS.recal” in dashed blue)

In NL data, both COPE and NOCOS had a negative net benefit for decision thresholds over approximately 30% (Fig. 4A). For thresholds below this level, the net benefit of COPE was generally better than that of NOCOS. Surprisingly, recalibration of both models to the second-wave NL data led to worse net benefit in this range, probably because linear recalibration of the intercept and slope was insufficient. In the NYC data, the net benefit of COPE was negative for decision thresholds over 15% and clearly improved after recalibration of the intercept and the slope to the second-wave NYC data (Fig. 4B). The net benefit of NOCOS was positive for the full range of decision thresholds in the NYC data, and did not benefit from recalibration because NOCOS was already well calibrated in second-wave NYC data. After recalibration, the decision curves of COPE and NOCOS were quite similar.

## Discussion

We examined the performance of prognostic models developed on the “first wave” COVID-19 data to predict mortality during the second wave, both locally and in a different setting. The model developed in the Netherlands (COPE) had reasonably good performance in both settings, except with some over-prediction of risk in the NYC area. This performance was only achieved by carefully modeling the effect of secular changes during the first wave such that predictions were calibrated to yield risks consistent with the end of the first wave (August 2020). The model developed in the NYC area (NOCOS), greatly over-predicted risk in both NL and in NYC during the second wave, despite including a variable to capture the effect of calendar time in the first wave. These results underscore the need for caution when transporting prognostic models over time and space: sometimes these models work, but sometimes they don’t — and specifics matter. In particular, we observed that calibration may be especially sensitive to changes in setting, consistent with our prior work [11, 21].

It is unsurprising that models developed on data from March 2020 at the very beginning of the pandemic led to profound over-prediction of mortality risk during the second wave. Presumably, this in part reflects a “learning curve” as clinical management evolved rapidly over time. This might be due to the development of specific therapeutic approaches – including proning, minimizing paralytics, changes in ventilator volume settings, remdesivir, dexamethasone, and other treatments — as well as general improvements in supportive care, which may relate to the capacity of the health systems to cope with overwhelming volumes. Based on our findings, it appears that the “first wave effect” was more prolonged in NYC, since accounting for the secular trend within the first wave improved COPE predictions substantially but not NOCOS predictions. Again, this might be anticipated given the intensity of the pandemic in this region. Despite less than excellent performance on second-wave data, decision curve analysis generally showed positive net benefit across most thresholds, except at high-risk levels above which there were few patients.

An interesting finding from the models predicting the need for mechanical ventilation is that they both appeared to be under-fit, with stronger predictor effects (slope > 1.0) in second-wave data and a “paradoxical” improvement in discriminative ability on validation data. This suggests that mechanical ventilation might have been better targeted to patients at higher risk in the second wave in both settings.

A recent systematic review examined more than 200 COVID-19 models and found that these did not generally apply rigorous development methods [7]. All reviewed models demonstrated a high or unclear risk of bias when evaluated using the prediction model risk of bias assessment tool (PROBAST) [22]. We specifically used PROBAST as a guide when developing our model, to ensure methods consistent with a low risk of bias. Nevertheless, our results point to fundamental challenges of prediction when developing models during a dynamic pandemic, even when carefully adhering to good methodological practice. Techniques for dynamic updating of models may be needed in such circumstances [23–27]. While some of these challenges may be unique to COVID-19, recently the risk of poor model performance and the need for continual updating to avoid the potential for harmful decision-making has gotten increasing attention [9, 23, 26–30].

We note there are several limitations to our study. Performance of these models as measured here in second-wave data may not currently apply, since the pandemic has continued to evolve. In particular, the widespread dissemination of vaccines may well affect clinical presentation, patient risk, and predictor effects. So-called “breakthrough” COVID generally has a much lower mortality rate. These issues only strengthen the importance of our methodological conclusions. Another limitation is that we were limited to using variables that were routinely collected in both locations. In particular, we were limited to using variables present on ED presentation, which limited the performance of models used to predict outcomes in the subset of patients admitted to the ICU or placed on mechanical ventilation. Finally, the use of similar regression modeling strategies at both geographical sites may be considered a limitation.

Other investigators have underscored the fact that COVID-19 has posed many challenges for mathematical modelers, in particular, accurate forecasting of the pandemic has proven elusive [31]. Our findings underscore that the prediction of COVID-19 clinical outcomes may also have important challenges, since outcomes risks can be affected by variables that are not included in the model and that change over time and space, affecting the baseline risk and also modifying the effect of predictor variables in the model. In particular, mortality rates early in the epidemic were substantially higher than those later in the epidemic and this change over time was different across the two settings we examined. Additionally, improved targeted of mechanical ventilation over time led to paradoxically improved discrimination, although with poor calibration. These concerns point to the importance of dynamic model updating, which may need to be tailored to local circumstances, placing limits on the generalizability of global models.

We note that our study had several unique strengths. The databases used for model development were among the largest first-wave databases, including over 12,000 hospitalized patients from the NYC region and over 5000 hospitalized Dutch patients. They were both developed on multiple hospitals, also permitting rigorous internal-external validation approaches on model development and presumably improving model generalizability [32]. Unlike most prior models developed for in-hospital COVID-19 prognosis, we carefully adhered to methodological practices shown to be associated with a lower risk of bias [7, 33–35]. We used both conventional and novel measures of model performance, including decision curve analysis to assess clinical utility. Finally, ours is the only attempt we know of that has examined temporal and geographic validation of prognostic COVID-19 models in different pandemic waves and across different countries.

Future work should focus on methods for continuous dynamic model updating, including a comparison of different methods for updating [23, 25, 27, 36]. Furthermore, whether prognostic models improve process and clinical outcomes need to be studied, together with barriers and facilitators of their uptake in clinical practice.

## Conclusions

NOCOS performed moderately worse than COPE, both at temporal and geographic validation, likely reflecting unique aspects of the early pandemic in NYC. Frequent updating of prognostic models is likely to be required to for transportability over time and space during a dynamic pandemic.

## Supplementary Information

**Additional file 1: Box S1.** Description of the development details of COPE and NOCOS. **Table S1.** Regression model formulas of COPE and NOCOS.

## Data Availability

The datasets generated and/or analyzed during the current study are not publicly available due to data transfer agreements with the contributing hospitals but are available from the corresponding author on reasonable request.

## Abbreviations

ED: Emergency department
NYC: New York City
NOCOS: Northwell COVID-19 survival
COPE: COVID outcome prediction in the emergency department
AUC: Area under the operator receiver characteristic curve
ICU: Intensive care unit
CRP: C-reactive protein
LDH: Lactic dehydrogenase
MCV: Mean corpuscular volume
mb.c: Model-based concordance
PROBAST: Prediction model risk of bias assessment tool

## References

[1] Nkengasong J, Iwasaki A, Victora C, Oh J, Gao GF, Agrawal A (2020). The global response to the COVID-19 pandemic. Med (N Y).

[2] World Health Organization. Coronavirus disease (COVID-19) pandemic. Available from: https://www.who.int/health-topics/coronavirus. Accessed 18 Oct 2022.

[3] Richardson S, Hirsch JS, Narasimhan M, Crawford JM, McGinn T, Davidson KW (2020). Presenting characteristics, comorbidities, and outcomes among 5700 patients hospitalized with COVID-19 in the New York City area. JAMA..

[4] Zhou F, Yu T, Du R, Fan G, Liu Y, Liu Z (2020). Clinical course and risk factors for mortality of adult inpatients with COVID-19 in Wuhan, China: a retrospective cohort study. Lancet..

[5] Richardson S, Hirsch JS, Narasimhan M, Crawford JM, McGinn T, Davidson KW (2020). Clarification of mortality rate and data in abstract, results, and table 2. JAMA..

[6] Cazeau N (2020). Social Isolation: Managing Psychological Distress in Hospitalized Patients During the COVID-19 Pandemic. Clin J Oncol Nurs.

[7] Wynants L, Van Calster B, Collins GS, Riley RD, Heinze G, Schuit E (2020). Prediction models for diagnosis and prognosis of COVID-19: systematic review and critical appraisal. BMJ..

[8] Lombardi Y, Azoyan L, Szychowiak P, Bellamine A, Lemaitre G, Bernaux M (2021). External validation of prognostic scores for COVID-19: a multicenter cohort study of patients hospitalized in Greater Paris University Hospitals. Intensive Care Med.

[9] Van Calster B, McLernon DJ, van Smeden M, Wynants L, Steyerberg EW (2019). Calibration: The Achilles heel of predictive analytics. BMC Med.

[10] Van Calster B, Vickers AJ (2015). Calibration of risk prediction models: impact on decision-analytic performance. Med Decis Mak.

[11] Gulati G, Upshaw J, Wessler BS, Brazil RJ, Nelson J, van Klaveren D (2022). Generalizability of cardiovascular disease clinical prediction models: 158 independent external validations of 104 unique models. Circ Cardiovasc Qual Outcomes.

[12] Barouch DH (2022). COVID-19 vaccines — immunity, variants, boosters. N Engl J Med.

[13] COVID-19 Treatment Guidelines Panel. Coronavirus Disease 2019 (COVID-19) Treatment Guidelines. National Institutes of Health. Available at https://www.covid19treatmentguidelines.nih.gov/. Accessed 18 October 2022.

[14] Levy TJ, Richardson S, Coppa K, Barnaby DP, McGinn T, Becker LB, et al. Development and validation of a survival calculator for hospitalized patients with COVID-19. medRxiv. 2020:2020.04.22.20075416. 10.1101/2020.04.22.20075416.

[15] van Klaveren D, Rekkas A, Alsma J, Verdonschot R, Koning D, Kamps MJA (2021). COVID outcome prediction in the emergency department (COPE): using retrospective Dutch hospital data to develop simple and valid models for predicting mortality and need for intensive care unit admission in patients who present at the emergency department with suspected COVID-19. BMJ Open.

[16] R Core Team (2020). R: A language and environment for statistical computing. R Foundation for Statistical Computing, Vienna, Austria. 2020. URL http://www.R-project.org/.

[17] Van Buuren S, Groothuis-Oudshoorn K (2011). MICE: Multivariate Imputation by Chained Equations in R. J Stat Softw.

[18] van Klaveren D, Gonen M, Steyerberg EW, Vergouwe Y (2016). A new concordance measure for risk prediction models in external validation settings. Stat Med.

[19] Harrell FE (2001). Regression Modeling Strategies: With Applications to Linear Models, Logistic Regression, and Survival Analysis.

[20] Vickers AJ, Elkin EB (2006). Decision curve analysis: a novel method for evaluating prediction models. Med Decis Mak.

[21] Wessler BS, Nelson J, Park JG, McGinnes H, Gulati G, Brazil R (2021). External Validations of Cardiovascular Clinical Prediction Models: A Large-Scale Review of the Literature. Circ Cardiovasc Qual Outcomes.

[22] Moons KGM, Wolff RF, Riley RD, Whiting PF, Westwood M, Collins GS (2019). PROBAST: A Tool to Assess Risk of Bias and Applicability of Prediction Model Studies: Explanation and Elaboration. Ann Intern Med.

[23] Schnellinger EM, Yang W, Kimmel SE (2021). Comparison of dynamic updating strategies for clinical prediction models. Diagn Progn Res.

[24] Su TL, Jaki T, Hickey GL, Buchan I, Sperrin M (2018). A review of statistical updating methods for clinical prediction models. Stat Methods Med Res.

[25] Siregar S, Nieboer D, Vergouwe Y, Versteegh MI, Noyez L, Vonk AB (2016). Improved Prediction by Dynamic Modeling: An Exploratory Study in the Adult Cardiac Surgery Database of the Netherlands Association for Cardio-Thoracic Surgery. Circ Cardiovasc Qual Outcomes.

[26] Davis SE, Greevy RA, Lasko TA, Walsh CG, Matheny ME (2020). Detection of calibration drift in clinical prediction models to inform model updating. J Biomed Inform.

[27] Chi S, Tian Y, Wang F, Zhou T, Jin S, Li J (2022). A novel lifelong machine learning-based method to eliminate calibration drift in clinical prediction models. Artif Intell Med.

[28] Gulati G, Upshaw JN, Wessler BS, Brazil RJ, Nelson J, van Klaveren D, et al. The Generalizability of Cardiovascular Disease Clinical Prediction Models: 158 Large-Scale Independent External Validations of 104 Unique Models. Circ Cardiovasc Qual Outcomes. 2022;15(4):e008487. 10.1161/CIRCOUTCOMES.121.008487 PMC9015037 35354282

[29] Shah N, Steyerberg E, Kent D (2018). Big Data and Predictive Analytics: Recalibrating Expectations. JAMA..

[30] Jenkins DA, Martin GP, Sperrin M, Riley RD, Debray TPA, Collins GS (2021). Continual updating and monitoring of clinical prediction models: time for dynamic prediction systems?. Diagn Progn Res.

[31] Ioannidis JPA, Cripps S, Tanner MA (2022). Forecasting for COVID-19 has failed. Int J Forecast.

[32] Steyerberg EW, Harrell FE (2016). Prediction models need appropriate internal, internal-external, and external validation. J Clin Epidemiol.

[33] Wolff RF, Moons KGM, Riley RD, Whiting PF, Westwood M, Collins GS (2019). PROBAST: A Tool to Assess the Risk of Bias and Applicability of Prediction Model Studies. Ann Intern Med.

[34] Venema E, Wessler BS, Paulus JK, Salah R, Raman G, Leung LY (2021). Large-scale validation of the Prediction model Risk Of Bias ASsessment Tool (PROBAST) using a short form: high risk of bias models show poorer discrimination. J Clin Epidemiol.

[35] Helmrich I, Mikolic A, Kent DM, Lingsma HF, Wynants L, Steyerberg EW (2022). Does poor methodological quality of prediction modeling studies translate to poor model performance? An illustration in traumatic brain injury. Diagn Progn Res.

[36] Hickey GL, Grant SW, Caiado C, Kendall S, Dunning J, Poullis M (2013). Dynamic prediction modeling approaches for cardiac surgery. Circ Cardiovasc Qual Outcomes.

