# Supplementary material for: Prognostic models for COVID-19 needed updating to warrant transportability over time and space
Source: BMC Med. 2022 Nov 23;20:456. doi: 10.1186/s12916-022-02651-3 (PMC9686462; doi:10.1186/s12916-022-02651-3)
Supplement: Supplementary file 1 — Additional file 1: Box S1. Description of the development details of COPE and NOCOS. Table S1. Regression model formulas of COPE and NOCOS. [file 12916_2022_2651_MOESM1_ESM.docx]

**Box S1 Description of the development details of COPE and NOCOS**

| For the development of COPE, the most promising predictors were selected into a parsimonious model – based on Wald test statistics of a multivariable model containing all 22 potential predictors – and bootstrap validation of the modeling strategy, including predictor selection, was used to shrink predicted probabilities to the average. COPE was developed on first-wave data to predict death within 28 days after hospital admission. To obtain robust predictions of the need for ICU admission, COPE was calibrated to this outcome, that is the intercept and the slope of the mortality model were updated based on the observed need for ICU admission in the first wave of the pandemic.  For the development of NOCOS, least absolute shrinkage and selection operator (LASSO) regression was used to select the strongest 6 out of 22 potential predictors. The same candidate predictors were used to accommodate validation of models across geographic sites. NOCOS was originally developed on early first-wave data (between March 1 and May 6, 2020) to predict death within 7 days after hospital admission. Here, we used the full first-wave data to adapt NOCOS for predicting 28-day outcomes: death and requiring mechanical ventilation. |
| --- |

**Table S1 Regression model formulas of COPE and NOCOS**Age = Age(years); RR = Respiratory rate (/min); CRP = C-Reactive protein (mg/L); LDH = Lactate dehydrogenase (U/L); Albumin = Serum Albumin (g/L); Urea = Serum Urea (mmol/L); Saturation = Oxygen saturation (%); Male = Male sex; Temperature = Body temperature (°C); D.dimer = D dimer (µg/L); log = natural logarithm; exp = natural exponential. For conversion of NL measurement units to NYC measurement units, we used: Blood Urea Nitrogen (mg/dl) = Urea (mmol/l) / 0.357; Bromocresol purple determined Serum Albumin (g/dL) = Immunochemically determined Serum Albumin (g/L) / 11.7.

| Outcome | COPE | NOCOS |
| --- | --- | --- |
| Death within 28 days | lp_D_ =  -13.6 +  0.04575 × Age +  1.654 × log(RR) +  0.1688 × log(CRP) +  1.197 × log(LDH) +  -1.585 × log(Albumin) +  0.5953 × log(Urea)  Probability = 1 / (1 + exp( -lp )) | lp_D_ =  -8.322 +  0.02746 × Age +  0.001951 × RR +  0.1435 × log(CRP) +  0.6946 × log(LDH) +  -0.1170 × (Albumin/11.7) +  0.5999 × log(Urea/.357) +  -0.01545 × Saturation  Probability = 1 / (1 + exp( -lp )) |
| ICU admission (COPE) or on ventilation (NOCOS) within 28 days | Probability =  1 / (1 + exp(-( -0.08949 + 0.5970 × lp_D_ ))) | lp_ICU_ =  -7.670 +  0.003269 × RR +  0.2566 × log(CRP) +  1.067 × log(LDH) +  0.06290 × log(Urea/.357) +  -0.02842 × Saturation +  0.2085 × Male +  0.003639 × Temperature  Probability = 1 / (1 + exp( -lp_ICU_)) |
